# Regulation of Antimycin Biosynthesis Is Controlled by the ClpXP Protease

**DOI:** 10.1128/mSphere.00144-20

**Published:** 2020-04-08

**Authors:** Bohdan Bilyk, Sora Kim, Asif Fazal, Tania A. Baker, Ryan F. Seipke

**Affiliations:** a Astbury Centre for Structural Molecular Biology, Faculty of Biological Sciences, University of Leeds, Leeds, United Kingdom; b Department of Biology, Massachusetts Institute of Technology, Cambridge, Massachusetts, USA; c Howard Hughes Medical Institute, Chevy Chase, Maryland, USA; University of Iowa

**Keywords:** ClpXP, ECF sigma factors, *Streptomyces*, antimycin, proteolysis, regulation of secondary metabolism

## Abstract

The survival of any microbe relies on its ability to respond to environmental change. Use of extracytoplasmic function (ECF) RNA polymerase sigma (σ) factors is a major strategy enabling dynamic responses to extracellular signals. *Streptomyces* species harbor a large number of ECF σ factors, nearly all of which are uncharacterized, but those that have been characterized generally regulate genes required for morphological differentiation and/or response to environmental stress, except for σ^AntA^, which regulates starter-unit biosynthesis in the production of antimycin, an anticancer compound. Unlike a canonical ECF σ factor, whose activity is regulated by a cognate anti-σ factor, σ^AntA^ is an orphan, raising intriguing questions about how its activity may be controlled. Here, we reconstituted *in vitro* ClpXP proteolysis of σ^AntA^ but not of a variant lacking a C-terminal di-alanine motif. Furthermore, we show that the abundance of σ^AntA^ *in vivo* was enhanced by removal of the ClpXP recognition sequence and that levels of the protein rose when cellular ClpXP protease activity was abolished. These data establish direct proteolysis as an alternative and, thus far, unique control strategy for an ECF RNA polymerase σ factor and expands the paradigmatic understanding of microbial signal transduction regulation.

**IMPORTANCE** Natural products produced by *Streptomyces* species underpin many industrially and medically important compounds. However, the majority of the ∼30 biosynthetic pathways harbored by an average species are not expressed in the laboratory. This unrevealed biochemical diversity is believed to comprise an untapped resource for natural product drug discovery. Major roadblocks preventing the exploitation of unexpressed biosynthetic pathways are a lack of insight into their regulation and limited technology for activating their expression. Our findings reveal that the abundance of σ^AntA^, which is the cluster-situated regulator of antimycin biosynthesis, is controlled by the ClpXP protease. These data link proteolysis to the regulation of natural product biosynthesis for the first time to our knowledge, and we anticipate that this will emerge as a major strategy by which actinobacteria regulate production of their natural products. Further study of this process will advance understanding of how expression of secondary metabolism is controlled and will aid pursuit of activating unexpressed biosynthetic pathways.

## Abstract (precis)

Natural products produced by *Streptomyces* species underpin many industrially and medically important compounds. However, the majority of the ∼30 biosynthetic pathways harbored by an average species are not expressed in the laboratory. This unrevealed biochemical diversity is believed to comprise an untapped resource for natural product drug discovery. Major roadblocks preventing the exploitation of unexpressed biosynthetic pathways are a lack of insight into their regulation and limited technology for activating their expression. Our findings reveal that the abundance of σ^AntA^, which is the cluster-situated regulator of antimycin biosynthesis, is controlled by the ClpXP protease. These data link proteolysis to the regulation of natural product biosynthesis for the first time to our knowledge, and we anticipate that this will emerge as a major strategy by which actinobacteria regulate production of their natural products. Further study of this process will advance understanding of how expression of secondary metabolism is controlled and will aid pursuit of activating unexpressed biosynthetic pathways.

## INTRODUCTION

The survival of any organism relies on its ability to respond to environmental change. This feature is especially true of bacteria, which often live in hostile and fluctuating environments. *Streptomyces* bacteria thrive in soils. The success of this genus of filamentous, sporulating bacteria is linked to their complex life cycle and keen ability to sense and respond to the surroundings. Notably, a multitude of bioactive secondary or specialized metabolites are produced in response to environmental cues (1). More than half of all small-molecule therapeutics critical for human health and well-being are derived from or inspired by *Streptomyces* natural products (2).

*Streptomyces* species typically harbor a large number of biosynthetic pathways, but only a few of them are expressed under common laboratory conditions. The biochemical diversity encoded by these silent pathways is a tremendous untapped resource for discovery of new antibacterial agents and other therapeutics. All available data indicate that the production of natural products is controlled predominantly at the level of transcription. Although there are complex regulatory cascades that tightly control expression of biosynthetic genes, they are ultimately activated, repressed, or derepressed by so-called cluster-situated regulators—regulatory proteins encoded within the biosynthetic gene cluster (BGC) (3, 4). Major roadblocks preventing the exploitation of silent biosynthetic pathways are a lack of insight into their regulation and limited technology for activating their expression.

**FIG 1:**
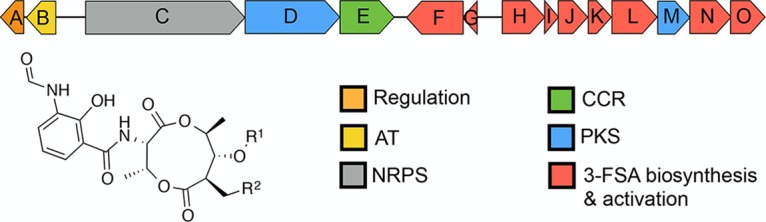
Schematic representation of the antimycin (*ant*) biosynthetic gene cluster. AT, acyltransferase; NRPS, nonribosomal peptide synthetase; PKS, polyketide synthase; CCR, crotonyl coenzyme A (crotonyl-CoA) carboxylase/reductase; 3-FSA, 3-formamidosalicylate. Antimycins: antimycin A_1_, R^1^= COCH(CH_3_)CH_2_CH_3_, R^2^= (CH_2_)_4_CH_3_; antimycin A_2_, R^1^=COCH(CH_3_)_2_, R^2^= (CH_2_)_4_CH_3_; antimycin A_3_, R^1^= COCH_2_CH(CH_3_)_2_, R^2^= (CH_2_)_2_CH_3_; antimycin A_4_, R^1^= COCH(CH_3_)_2_, R^2^= (CH_2_)_2_CH_3_.

Antimycins have been known for 70 years and are the founding members of a large class of natural products widely produced by *Streptomyces* species (5, 6). Recently, antimycins were shown to be potent and selective inhibitors of the mitochondrial Bcl-2/Bcl-X_L_-related antiapoptotic proteins that are overproduced by cancer cells and confer resistance to chemotherapeutic agents whose mode of action is activation of apoptosis (7). The ∼25-kb antimycin (*ant*) BGC harbored by Streptomyces albus is composed of 15 genes organized into four polycistronic operons: *antBA*, *antCDE*, *antGF*, and *antHIJKLMNO* (Fig. 1) (8, 9). The regulation of the *ant* BGC is unusual compared to other secondary metabolites. Its expression is regulated by FscRI, a cluster-situated LuxR-family regulator of candicidin biosynthesis; FscRI activates expression of *antBA* and *antCDE* (10). Importantly, *antA* is a cluster-situated regulator that encodes an extracytoplasmic function (ECF) RNA polymerase σ factor (σ^AntA^) that activates expression of the remaining operons: *antGF* and *antHIJKLMNO* (Fig. 1) (9).

σ^AntA^, like all ECF σ factors, is similar to members of the housekeeping σ^70^ family but possesses only two of the four highly characteristic sigma domains: domains σ2 and σ4. The σ2 and σ4 regions of sigma factors bind the −10 and −35 promoter elements, respectively, and are sufficient for recruitment of RNA polymerase (11). Genes encoding ECF σ factors are almost always cotranscribed with their cognate anti-σ factor (12). Anti-σ factors are generally transmembrane proteins, but well-characterized examples of cytoplasmic anti-σ factors are known (13–15). Anti-σ factors selectively bind to and sequester a partner σ factor until its release is stimulated, usually by an exogenous signal (12, 16). After the σ factor is released, it recruits RNA polymerase to express a defined regulon that usually includes the σ factor–anti-σ factor operon itself, which thus establishes a positive auto-feedback loop in the presence of the inducing stimulus. Even when an ECF σ factor does have a cognate anti-σ factor, an additional mechanism of control can also exist, for instance, σ^Rʹ^ in S. coelicolor is processed by the Clp-protease system (17). *Streptomyces* species encode a large number of ECF σ factors (>30 per strain), and the small number of these that have been characterized regulate genes required for morphological differentiation and/or responses to environmental stress and are not dedicated regulators of one biosynthetic pathway. Indeed, cluster-situated ECF σ factors have been observed previously only in the biosynthesis of lantibiotics produced by so-called rare actinomycetes. In Microbispora corallina, MibR and σ^MibX^ regulate microbisporicin biosynthesis and σ^MibX^ is controlled by the anti-σ factor MibW (18); in Planomonospora alba, PspR and σ^PspX^ regulate planosporicin production and σ^PspX^ is controlled by the anti-σ factor PspW (19). Interestingly, unlike the canonical ECF σ factors σ^MibX^ and σ^PspX^, whose activities are controlled by cognate anti-σ factors, σ^AntA^ lacks an identifiable anti-σ factor partner and as a consequence has created curiosity about how its activity is controlled.

The Clp-protease system is essential for normal bacterial proteostasis and is best characterized in Escherichia coli (20, 21). The Clp protease is a multienzyme complex composed of a barrel-shaped peptidase, ClpP, and a regulatory enzyme, either ClpA or ClpX (or ClpC in some organisms). ClpA and ClpX (and ClpC) are AAA+-family protein unfoldases that recognize an N-terminal and/or C-terminal recognition signal (degron) and utilize ATP to unfold and translocate proteins to the peptidase chamber, where they are degraded into short peptides (22). In *Streptomyces* species, the peptidase is specified by two genes instead of one and is redundantly encoded (23). The primary peptidase is encoded by *clpP1P2*, whose corresponding proteins form a complex with ClpX or ClpA to facilitate normal proteostasis; the second peptidase is encoded by *clpP3P4*, but its expression occurs only when the primary system is compromised (24, 25). The best-understood degron is the SsrA tag from E. coli ( AANDENYALAA), which is added cotranslationally to polypeptides stalled on ribosomes (26, 27). The E. coli SsrA tag has been comprehensively studied, and Ala-Ala-COO-, at the C-terminal region of this motif, is essential for proteolysis by ClpXP (28). Intriguingly, the C terminus of σ^AntA^ harbors the sequence Ala-Ala-COO-, which previously led us to speculate that ClpXP may modulate its level/activity (9).

Here, we reconstituted ClpXP proteolysis of σ^AntA^ *in vitro* and showed that it is dependent upon the C-terminal Ala-Ala. We also found that the abundance of σ^AntA^ *in vivo* was higher when Ala-Ala was changed to Asp-Asp and that the abundance of σ^AntA^ was elevated in the absence of genes encoding the primary peptidase, ClpP, and its unfoldase, ClpX. These data establish direct proteolysis as an alternative, and thus far unique, control strategy of ECF RNA polymerase σ factors, expanding the paradigmatic understanding of microbial signal transduction regulation.

## RESULTS AND DISCUSSION

### σ^AntA^ orthologues are a new subfamily of ECF σ factors that regulate production of the antimycin biosynthetic starter unit.

**FIG 2:**
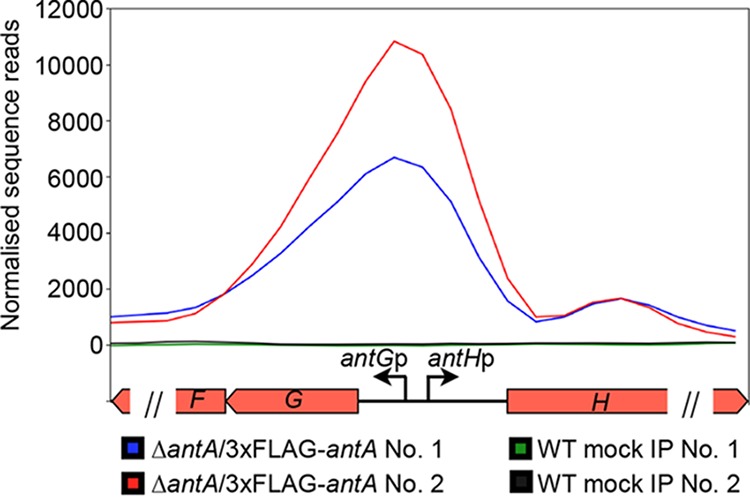
3xFLAG-σ^AntA^ binds to the *antGF* and *antHIJKLMNO* promoters *in vivo.* Shown is a graphical representation of normalized sequence reads mapped to the intergenic region of *antG-antH* (shown at the bottom). The 1,242-bp genomic window depicts nucleotides 34,430 to 35,671 of contig CADY01000091.1 of the S. albus S4 genome. Each double slash denotes that genome window presented does not contain the entire *antF* or *antH* coding sequence. WT, wild type; IP, immunoprecipitation.

Since its initial discovery 6 years ago, more than 70 *ant* BGCs have been identified within the members of *Actinobacteria*, including *Actinospica*, *Saccharopolyspora*, *Streptacidiphilus*, and *Streptomyces* (5). Each of these BGCs harbors a single regulator, σ^AntA^ (53% to 100% shared amino acid identity across all orthologues), which lacks a cognate anti-σ factor partner (5, 9). Our previous work with S. albus S4 established that σ^AntA^ orthologues comprise a new subfamily of ECF σ factors (9, 29). We demonstrated that σ^AntA^ is required for expression of *antGF* and *antHIJKLMNO*, which encode a standalone ketoreductase (AntM) and proteins required for the production/activation of the starter unit, 3-formamidosalicylate (3-FSA) (Fig. 1). We also mapped the transcriptional start sites and identified conserved promoter sequences for these operons in all *ant* BGCs known at the time (9). The conservation of σ^AntA^ and target promoters within *ant* BGCs from taxonomically diverse species suggests that σ^AntA^-mediated regulation of these genes is direct. To verify this hypothesis, we performed chromatin immunoprecipitation sequencing (ChIP-seq) with a S. albus S4 Δ*antA* mutant complemented with a version of σ^AntA^ with a 3xFLAG tag at its N terminus, which we demonstrated restored antimycin production (see Fig. S2 in the supplemental material). Analysis of the resulting data revealed only one ChIP-seq peak across the whole chromosome for which the number of mapped reads was enriched for both biological replicates of Δ*antA*/3xFLAG-*antA* compared to that of the wild-type mock-immunoprecipitated control. This region corresponded to the intergenic space (297 bp) between *antG* and *antH*, which upon inspection revealed a prominent peak for the closely spaced and divergent σ^AntA^-target promoters *antG*p and *antH*p and a second, albeit smaller peak corresponding to the 5′ end of the *antH* coding sequence (Fig. 2). Taken together, these data are consistent with the hypothesis that σ^AntA^ is a cluster-situated regulator that directly activates expression of genes for the production of 3-FSA during antimycin biosynthesis.

### σ^AntA^ is degraded by the ClpXP protease *in vitro*.

**FIG 3:**
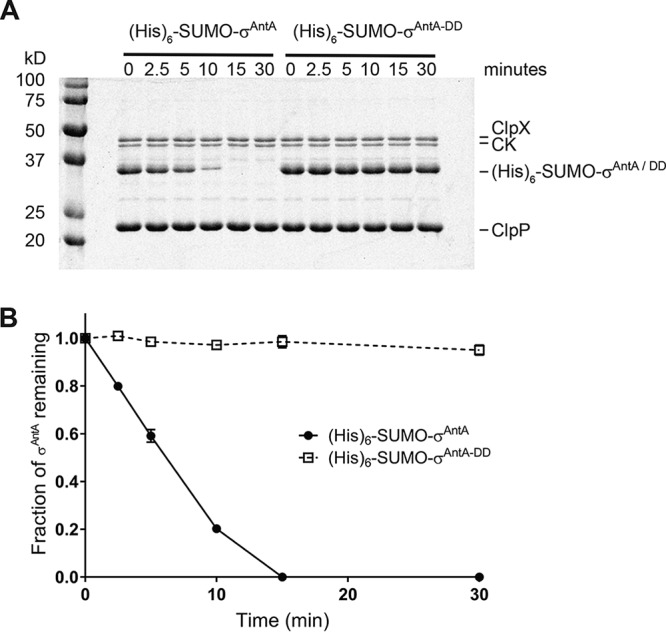
Proteolysis of S. ambofaciens σ^AntA^ by ClpXP *in vitro*. (A) SDS-PAGE analysis of proteolysis reaction mixtures containing 37 pmol (His)_6_SUMO-σ^AntA^ or (His)_6_SUMO-σ^AntA-DD^. (B) Densitometry analysis SDS-PAGE images for three independent proteolysis experiments. The mean is plotted, and error bars illustrate the standard error of the mean (±1 SEM).

The activities of almost all characterized ECF σ factors are modulated by a cognate anti-σ factor, which is typically a protein coencoded within the same operon. Intriguingly, σ^AntA^ lacks an anti-σ factor and is therefore an orphan, indicating that a unique mechanism is likely at work to control σ^AntA^ activity. An inspection of σ^AntA^ amino acid sequences revealed a C-terminal Ala-Ala in 67 of the 71 orthologues (Fig. S1). A C-terminal Ala-Ala is an important component of a common class of degrons for the ClpXP protease (28). This observation led us to hypothesize that the activity of σ^AntA^ might be modulated by proteolysis instead of by an anti-σ factor. To test this hypothesis, we performed *in vitro* proteolysis. Previous work indicated that S. albus S4 σ^AntA^ was insoluble when overproduced by E. coli, so we pursued the overproduction and purification of the orthologue from Streptomyces ambofaciens ATCC 23877, which has been experimentally demonstrated to be a producer of antimycins (30). S. ambofaciens σ^AntA^ (75% amino acid identity with S. albus S4 σ^AntA^, including 13 of 15 amino acid residues at the C terminus) was purified as an N-terminal (His)_6_-SUMO-fusion protein. The (His)_6_-SUMO tag increases solubility and eases purification of putative substrates, without altering recognition of C-terminal degrons by ClpXP. ClpX orthologues from E. coli and S. ambofaciens possess 60% shared amino acid identity and therefore likely recognize similar substrates for degradation. Thus, ClpXP from E. coli was purified (Fig. S2) and its ability to degrade (His)_6_-SUMO-σ^AntA^ was assessed. Degradation of (His)_6_-SUMO-σ^AntA^ was apparent as early as 2.5 min after addition of ATP, and all of the sample was degraded by 15 min (Fig. 3). Substrates of ClpXP become resistant to proteolysis by specific alterations of the C-terminal Ala-Ala (28). Therefore, to investigate degradation specificity in the experiment described above, we constructed and tested a variant of S. ambofaciens σ^AntA^ in which the C-terminal Ala-Ala was mutated to Asp-Asp [(His)_6_-SUMO-σ^AntA-DD^]. Strikingly, the Asp-Asp variant was stable against ClpXP degradation over the lifetime of the assay (Fig. 3). Thus, the degradation of (His)_6_-SUMO-σ^AntA^ and the characteristic resistance afforded by the Ala-Ala-to-Asp-Asp mutation demonstrated that σ^AntA^ is a substrate of ClpXP *in vitro*.

10.1128/mSphere.00144-20.1 FIG S1 MUSCLE alignment of 71 σ^AntA^ orthologues. Only alignments of amino acid residues 160 to 180 are shown. Red asterisks indicate the C-terminal Ala-Ala motif conserved in 67 of 71 orthologues. Blue shading indicates the names of those taxa that do not possess the motif. The orthologue from *Streptomyces* sp. strain NRRL B-2790 terminated in Ala-Ala-Tyr; the inclusion of a terminal Tyr residue likely represents an artefact caused by poor genome sequence quality (>4,000 contigs). The remaining three orthologues lacking the di-alanine motif are Streptacidiphilus albus strains (terminating in Val-Ala) and *Streptomyces* sp. strain URHA-0041 (Tyr-Gly). Download FIG S1, GIF file, 0.8 MB. Copyright © 2020 Bilyk et al. 2020 Bilyk et al. This content is distributed under the terms of the Creative Commons Attribution 4.0 International license.

10.1128/mSphere.00144-20.2 FIG S2 LC-HRMS analysis of Δ*antA*/pAU3-45-3xFLAG-*antA* strains. The *m*/*z* values corresponding to the [M + H]^+^ ions derived from antimycins A1 to A4 are shown. Download FIG S2, GIF file, 0.3 MB. Copyright © 2020 Bilyk et al. 2020 Bilyk et al. This content is distributed under the terms of the Creative Commons Attribution 4.0 International license.

### σ^AntA^ is degraded by the ClpXP protease *in vivo*.

**FIG 4:**
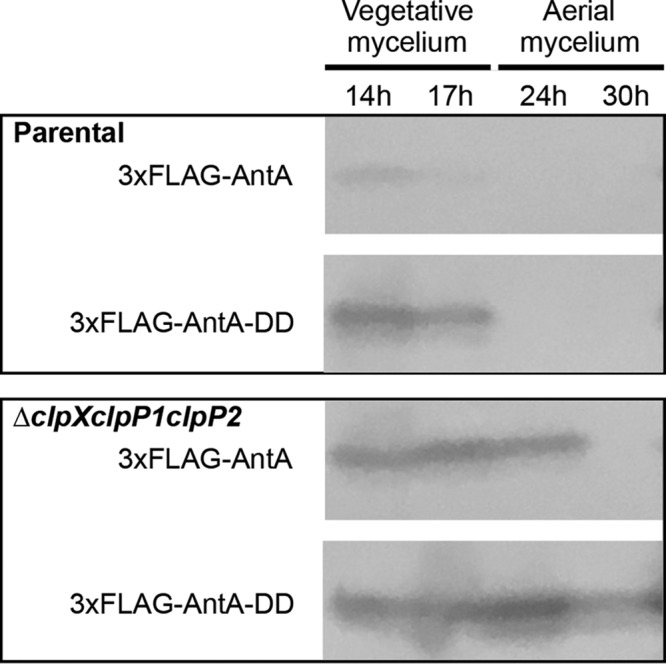
The abundance of σ^AntA^ is enhanced in the absence of the ClpXP *in vivo.* Cells from the indicated strains were cultivated over a developmental time course atop cellophane discs on agar media. Protein was isolated from 100 mg of either vegetative mycelium (14 and 17 h) or aerial mycelium (24 and 30 h). Thirty-microgram volumes of total protein were analyzed by Western blotting with anti-FLAG antisera. The images shown are derived from uncropped original images shown in Fig. S4 in the supplemental material, and the corresponding densitometry analysis is shown in Fig. S6.

To investigate if the *in vitro* degradation of σ^AntA^ demonstrated above is relevant to its regulation *in vivo*, we deleted the operon consisting of the *clpX*, *clpP1*, *and clpP2* genes from S. albus S4. The resulting Δ*clpXclpP1clpP2* mutant still harbored the second Clp peptidase encoded by *clpP3clpP4* and underwent a normal developmental cycle, albeit sporulation was less robust, which is consistent with growth characteristics reported for mutation of equivalent genes in S. coelicolor (Fig. S3) (31). Next, genes encoding the 3xFLAG-σ^AntA^ or 3xFLAG-σ^AntA-DD^ fusion proteins were generated and introduced into the parental strain and the *ΔclpXclpP1clpP2* mutant so the abundance of these proteins could be assessed over a developmental time course by Western blotting with anti-FLAG antisera. This experiment was initially performed with the σ^AntA^ fusions integrated on the chromosome under the control of the native promoter. However, a reliable signal could not be detected for 3xFLAG-σ^AntA^ and only a trace amount of the Asp-Asp variant was observed, presumably indicating that the cellular level of σ^AntA^ is normally low because the native promoter is relatively weak. The experiment was therefore repeated with 3xFLAG-σ^AntA^ and 3xFLAG-σ^AntA-DD^ expression driven by a stronger, constitutive promoter, ermE* (32). Analysis of the resulting immunoblot revealed that 3xFLAG-σ^AntA-DD^ was more abundant than 3xFLAG-σ^AntA^ in vegetative mycelium of the parent and Δ*clpXclpP1clpP2* strains (Fig. 4; see also Fig. S5). Strikingly, in the later stages of development after aerial mycelium had formed (24 h and 30 h), 3xFLAG-σ^AntA^ and 3xFLAG-σ^AntA-DD^ were detected only in the Δ*clpXclpP1clpP2* strain and not the parent; the Asp-Asp variant was also present in greater relative abundance (Fig. 4), which was consistent with our previous experiments that showed that the *ant* BGC is downregulated at the level of transcription upon the onset of aerial growth (9). Interestingly, the conspicuous absence of 3xFLAG-σ^AntA^ and the presence of 3xFLAG-σ^AntA-DD^ in protein samples prepared from the latest time point sampled suggest the potential involvement of an additional degradative factor(s). Taken together, these data support the hypothesis that the levels of σ^AntA^, and thus its ability to activate gene expression of *antFGHIJKLMNO*, are modulated by the ClpXP protease.

10.1128/mSphere.00144-20.3 FIG S3 Gel filtration of purified (His)_6_-SUMO-σ^AntA^ proteins. Gel filtration chromatograms show the elution profiles of purified (His)_6_-SUMO-σ^AntA^ and (His)_6_-SUMO-σ^AntA-DD^ proteins (upper panel) and results of associated SDS-PAGE analyses (lower panels). The chromatogram of (His)_6_-SUMO-σ^AntA^ differs slightly from that of (His)_6_-SUMO-σ^AntA-DD^ because gel filtration of (His)_6_-SUMO-σ^AntA^ protein was performed in a reaction mixture containing 50 mM HEPES-KOH (pH 7.5), 150 mM KCl, 10% glycerol, and 1 mM DTT, which was later dialyzed into the storage buffer, whereas gel filtration of (His)_6_-SUMO-σ^AntA-DD^ was performed in the storage buffer. Download FIG S3, GIF file, 1.3 MB. Copyright © 2020 Bilyk et al. 2020 Bilyk et al. This content is distributed under the terms of the Creative Commons Attribution 4.0 International license.

10.1128/mSphere.00144-20.4 FIG S4 Sporulation of S. albus S4 *clp* mutants. Photographs were taken after 6 days of growth on the indicated medium. The Δ*clpXclpP1*Δ*clpP2* strain underwent a normal developmental cycle; however, sporulation was less robust on MS agar media than was seen with the parental strain. Download FIG S4, GIF file, 0.2 MB. Copyright © 2020 Bilyk et al. 2020 Bilyk et al. This content is distributed under the terms of the Creative Commons Attribution 4.0 International license.

10.1128/mSphere.00144-20.5 FIG S5 Uncropped Western blotting images. Shown are the original Western blots depicted in Fig. 4. Red boxes indicate cropped sections for the strains indicated. The molecular weight (Mw) of the protein marker (M) is shown. Plus and minus symbols on each blot indicate positive-testing strains (mutant strain Δ*clpXclpP1clpP2*/pPDD) and negative controls (parental strain Δantall), respectively. Download FIG S5, GIF file, 1.2 MB. Copyright © 2020 Bilyk et al. 2020 Bilyk et al. This content is distributed under the terms of the Creative Commons Attribution 4.0 International license.

10.1128/mSphere.00144-20.6 FIG S6 Densitometry analysis of Western blotting results. The mean intensities (in pixels) of relevant bands from the Western blots shown in Fig. 4 are displayed for the indicated strains and time points. The mean pixel intensity was determined using Photoshop v20.0.6, and values were normalized to the intensity of the Δ*clpXclpP1clpP2*/pPDD sample contained on each membrane. Download FIG S6, GIF file, 0.08 MB. Copyright © 2020 Bilyk et al. 2020 Bilyk et al. This content is distributed under the terms of the Creative Commons Attribution 4.0 International license.

### Antimycins are not overproduced in the absence of ClpXP.

The results of the experiments described above indicate that the cellular level of σ^AntA^ was more abundant in the absence of the ClpXP protease. In order to determine if an increased level of this transcription factor ultimately influenced the final production titer of antimycins, we used liquid chromatography–high-resolution mass spectrometry (LC-HRMS) to assess the abundance of antimycins in chemical extracts generated from the Δ*clpXclpP1clpP2* and parental strains grown atop a cellophane disk on mannitol-soya flour (MS) agar in triplicate. The extracted ion chromatograms representing antimycin A_1_, A_2_, A_3_, and A_4_ were used to determine the peak area for each compound, which was subsequently normalized based on the wet mycelium weight of the sample. Interestingly, the results indicated that the total levels of antimycin production by the Δ*clpXclpP1clpP2* mutant (15.57 arbitrary units [AU] ± 2.86) and the parental strain (16.59 AU ± 1.12) were not statistically significantly different (*P* value, 0.59) (see Table S1 in the supplemental material). This result is consistent with a previous experiment where overexpression of *antA* did not increase the titer of antimycins, because that experiment showed that it resulted in overexpression of only *antGF* and *antHIJKLMNO* (genes encoding the production of the AntG*-S-*3-formamidosalicylate starter unit) and not the remaining genes (*antABCDE*) in the BGC (9). This also presumably indicates that starter unit biosynthesis is not rate limiting for antimycin production.

10.1128/mSphere.00144-20.7 TABLE S1 LCMS quantification of antimycin production. Download Table S1, DOCX file, 0.02 MB. Copyright © 2020 Bilyk et al. 2020 Bilyk et al. This content is distributed under the terms of the Creative Commons Attribution 4.0 International license.

### Model for the regulation of antimycin biosynthesis.

**FIG 5:**
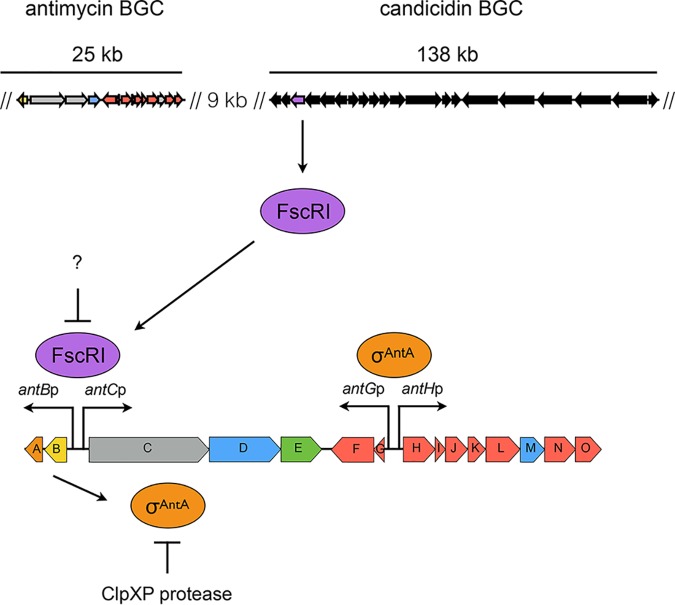
Model for the regulation of antimycin biosynthesis. The upper panel displays the relative locations of the antimycin and candicidin BGCs in the S. albus S4 chromosome. As shown in the lower panel, FscRI, a LuxR-family regulator, from the candicidin BGC, activates expression of *antBA* and *antCDE*. This in turn enables direct activation of the 3-FSA biosynthetic operons (*antGF* and *antHIJKLMNO*) by σ^AntA^. The cellular level of σ^AntA^ is antagonized by the ClpXP-protease system, for which it is a direct target and is ultimately responsible for clearing residual σ^AntA^ when FscRI is inactivated following the onset of differentiation.

Our model for the regulation of antimycin biosynthesis is depicted in Fig. 5. Expression of the *ant* BGC is cross-activated by FscRI, a LuxR-family regulator, from the candicidin BGC, which activates expression of *antBA* and *antCDE* (10). This regulation in turn enables direct activation of the 3-FSA biosynthetic operons (*antGF* and *antHIJKLMNO*) by σ^AntA^. The expression of *antBA* and *antCDE* is downregulated following the onset of morphological differentiation, presumably because the ligand sensed by the FscRI PAS domain is no longer available (9, 10). The cellular level of σ^AntA^ is antagonized by the ClpXP protease, for which it is a direct target, and is ultimately responsible for clearing residual σ^AntA^ when FscRI is inactivated following the onset of morphological differentiation (10). While ClpXP proteolytic control of transcription factor activity, and in particular that of ECF σ factor/anti-σ factors, has been shown previously (33–41), it has thus far not been directly linked to the control of cluster-situated regulators of natural product biosynthesis. This finding provides a new lens through which to examine microbial signal transduction and the regulation of natural product biosynthesis in *Streptomyces* species. Understanding the diversity of regulatory strategies controlling the expression of these pathways is critical for the development of new tools for exploiting the “silent majority” of biosynthetic pathways harbored by these organisms.

## MATERIALS AND METHODS

### Growth media, strains, cosmids, plasmids, and other reagents.

Escherichia coli strains were propagated on Lennox agar (LA) or broth (LB) (42, 43), and Streptomyces albus S4 strains were cultivated using LA, LB, and mannitol-soya flour (MS) agar or broth (42). Development of *clp* mutants was assessed on MS and ISP2 medium (42). Culture medium was supplemented with antibiotics as required at the following concentrations: apramycin, 50 μg ml^−1^; carbenicillin, 100 μg ml^−1^; chloramphenicol, 25 μg ml^−1^; hygromycin, 50 μg ml^−1^; kanamycin, 50 μg ml^−1^; nalidixic acid, 25 μg ml^−1^. *Streptomyces* strains were constructed by conjugal mating with E. coli ET12567 as previously described (42). Enzymes were purchased from New England Biolabs unless otherwise stated, and oligonucleotides were purchased from Integrated DNA Technologies, Inc. All of the strains, cosmids, and plasmids used in this study are described in Table S2 in the supplemental material, and all of the oligonucleotides used are provided in Table S3.

10.1128/mSphere.00144-20.8 TABLE S2 Bacterial strains, cosmids, and plasmids used in this study. Download Table S2, DOCX file, 0.02 MB. Copyright © 2020 Bilyk et al. 2020 Bilyk et al. This content is distributed under the terms of the Creative Commons Attribution 4.0 International license.

10.1128/mSphere.00144-20.9 TABLE S3 Oligonucleotides used in this study. Download Table S3, DOCX file, 0.02 MB. Copyright © 2020 Bilyk et al. 2020 Bilyk et al. This content is distributed under the terms of the Creative Commons Attribution 4.0 International license.

### Construction of plasmids.

The insertion for each plasmid generated in this study was prepared by PCR amplification with Q5 High-Fidelity DNA polymerase and oligonucleotides containing restriction sites. PCR-amplified insertions were restricted and cloned into the relevant plasmids cut with the same enzymes by standard molecular biology procedures. All clones were sequenced to verify the integrity of insertion DNA. The names of the restriction sites used for cloning are provided with the plasmid descriptions in Table S2.

### ChIP-seq and bioinformatics analyses.

The *antA* coding sequence was amplified with RFS629 and RFS630, which contain KpnI and EcoRI restriction sites, respectively. The restricted PCR product was cloned into pSETNFLAG (10) digested with the same enzymes. The resulting plasmid was then restricted with NotI and EcoRI to release ermE*p-3xFLAG-*antA*, which was subsequently cloned into pAU3-45 (44) digested with the same enzymes. pAU3-45-3xFLAG-*antA* was mobilized to an apramycin-marked Δ*antA* strain (9). Cultivations of the wild-type and Δ*antA*/pAUNFLAG-*antA* strains for ChIP-seq were performed exactly as described previously (10). Pure DNA resulting from immunoprecipitates from two biological replicates of the wild-type and Δ*antA*/pAUNFLAG-*antA* strains and nonimmunoprecipitated chromosomal DNA were sequenced with a Illumina HiSeq 3000 platform with 150-nucleotide (nt) paired-end reads by the University of Leeds Next Generation Sequencing Facility at the St. James Teaching Hospital NHS Trust. The resulting reads were analyzed exactly as described previously (10). The graphic in Fig. 2 was generated using DeepTools computeMatrix in scale-regions mode with a bin size of 54 and plotProfile functions (45).

### Construction of the S. albus S4 Δ*clpXclpP1clpP2* mutant strain.

Deletion of *clpXclpP1clpP2* was carried out using RecET recombineering in E. coli as follows. The *clpXclpP1clpP2*-containing cosmid, cos117, was obtained by screening a previously constructed S. albus S4 Supercos1 cosmid library (8) by PCR using oligonucleotides PBB001 and PBB002. Cos117 was mutagenized as required using E. coli recombineering with strain GB05-red (46) and a deletion cassette. The deletion cassette was generated by PCR from paac-apr-oriT (47) and consisted of the *aac(*3*)IV* apramycin resistance gene and a conjugal origin of transfer (*oriT*), which was flanked by ΦC31-*attL* and ΦC31-*attR* sites for excision of the cassette. The oligonucleotides used to generate deletion cassettes included 39 nt of homology upstream or downstream of the target open reading frame(s) and are listed in Table S3. The resulting PCR product was digested with DpnI, gel purified, and electroporated into arabinose-induced E. coli GB05-red harboring cos117. Transformants were screened for the presence of mutagenized cosmid by PCR using oligonucleotides listed in Table S3, and the integrity of the locus was verified by DNA sequencing. The mutagenized cosmid was electroporated into E. coli ET12567/pUZ8002 and mobilized to a strain of S. albus S4 harboring an entire antimycin BGC deletion (Δantall) (48) by conjugation as described previously (42). Transconjugants were screened for apramycin resistance and kanamycin sensitivity. The integrity of an apramycin-marked mutant was verified by PCR using the oligonucleotides listed in Table S3. The apramycin deletion cassette was subsequently excised from the chromosome by conjugal introduction of pUWLint31, which is a replicative plasmid with a temperature-sensitive origin of replication that expresses the ΦC31 integrase required for removal of the cassette (47). Transconjugants were screened for loss of apramycin resistance, and excision of the cassette was verified by polymorphic shift PCR and DNA sequencing of the product.

### Immunoblot analysis.

Spores of the parental strain and of S. albus Δantall and the Δ*clpXclpP1clpP2* mutant harboring pPDA or pPDD were grown on MS agar (buffered with 50 mM TES, pH 7.2) covered with cellophane discs. Protein was isolated from mycelium collected during growth at the following regular intervals: 14 h, 17 h, 24 h, and 30 h for the Δantall and Δ*clpXclpP1clpP2* mutants harboring 3xFLAG-AntA constructs and 17 h, 20 h, 23 h, and 30 h for the Δantall and Δ*clpXclpP1clpP2* mutants harboring the 3xFLAG-FscRI construct. Protein samples were generated as follows: 100-mg volumes of cells were resuspended in 200 μl lysis buffer (50 mM sodium phosphate buffer [pH 7.0]; 150 mM sodium chloride; 10 mg ml^−1^ lysozyme; cOmplete, Mini, EDTA-free protease inhibitors [Roche]; 100 mg of 0.1-mm-diameter glass beads [PowerLyzer]) and lysed by vortex mixing for 30 min at 2,000 rpm and 37°C, with a subsequent incubation for another 30 min at 37°C. The obtained suspension was centrifuged for 20 min at 20,000 × *g* at 18°C. Each clarified protein sample (30 μg) was subjected to SDS-PAGE and then transferred to a nitrocellulose membrane (pore size, 0.2 μm) for Western blot analysis. The membrane was probed with mouse monoclonal anti-FLAG M2-horseradish peroxidase (HRP) antibody (Sigma) (1:10,000, and the signals were detected using Pierce 1-Step Ultra TMB blotting solution (Thermo Scientific).

### Protein purification and *in vitro* ClpXP proteolysis assays.

The wild-type *antA* gene was PCR amplified and cloned into the AgeI and HindIII sites of the pET23b-SUMO vector, which harbors an N-terminal (His)_6_-SUMO tag (49). The plasmid for production of (His)_6_-SUMO-σ^AntA-DD^ was generated by site-directed mutagenesis (Agilent QuikChange) using primers listed in Table S3. (His)_6_-SUMO-σ^AntA^ and (His)_6_-SUMO-σ^AntA-DD^ were produced by E. coli Rosetta(DE3) (Novagen) grown in LB at 37°C until an optical density at 600 nm (OD_600_) of 0.5 was reached, followed by induction with 0.4 mM IPTG (isopropyl-β-d-thiogalactopyranoside) and growth at 18°C for 16 h. Cells were resuspended in 50 mM sodium phosphate (pH 8)–1 M NaCl–20 mM imidazole–10% glycerol–1 mM dithiothreitol (DTT) and lysed by the use of a French press at 28,000 lb/in^2^, followed by treatment with protease inhibitor cocktail set III (EDTA-free) (Calbiochem), and benzonase (Millipore Sigma). (His)_6_-SUMO-σ^AntA^ and (His)_6_-SUMO-σ^AntA-DD^ proteins were purified by nickel-nitrilotriacetic acid (Ni-NTA) affinity chromatography and Superdex-75 gel filtration and stored in 50 mM potassium phosphate (pH 6.8)–850 mM KCl–10% glycerol–1 mM DTT. E. coli ClpX and ClpP proteins were purified as described previously (50, 51).

*In vitro* ClpXP proteolysis assays were performed at 30°C by preincubating 0.3 μM ClpX_6_ and 0.8 μM ClpP_14_ with an ATP regeneration system (4 mM ATP, 50 μg ml^−1^ creatine kinase, 5 mM creatine phosphate) in 25 mM HEPES-KOH (pH 7.5)–20 mM KCl–5 mM MgCl_2_–10% glycerol–0.032% NP-40–0.2 mM DTT and adding substrate to initiate the reactions. Samples of each reaction mixture were taken at specific time points, and the reactions were stopped by addition of SDS-PAGE loading dye and boiling at 100°C before loading on Tris-Glycine-SDS gels. Bands were visualized by staining with colloidal Coomassie G-250 and quantified by ImageQuant (GE Healthcare) after scanning by Typhoon FLA 9500 (GE Healthcare). The (His)_6_-SUMO-σ^AntA^ fraction remaining was calculated by dividing the (His)_6_-SUMO-σ^AntA^ density at a given time point by the density at time zero and normalizing by ClpX density.

### Chemical analysis.

S. albus S4 strains were cultivated atop a cellophane disc on MS agar at 30°C for 7 days in triplicate. At the time of harvest, the cellophane disc containing mycelium was removed and the quantity of biomass was determined. Bacterial metabolites were extracted from both the mycelium and the “spent” agar for 1 h using 50 ml of ethyl acetate. Thirty-milliliter volumes of ethyl acetate were evaporated to dryness under conditions of reduced pressure, and the resulting residue was resuspended in 100% methanol (300 μl). Immediately prior to LC-HRMS analysis, methanolic extracts were centrifuged at 16,000 × *g* in a microcentrifuge tube for 5 min to remove insoluble material. Only the supernatant (3 μl) was injected into a Bruker Maxis Impact time of flight (TOF) mass spectrometer equipped with a Dionext Ultimate 3000 HPLC system as previously described (52). The peak area associated with the extracted ion chromatograms for antimycin A_1_, A_2_, A_3_, and A_4_ present in agar and mycelium extracts was determined and used to calculate the total level of antimycins produced for each replicate. These values were subsequently used to determine the arithmetic mean for total antimycin production for each strain. Statistical significance was assessed in Microsoft Excel by a homoscedastic Student's *t* test with a two-tailed distribution.

### Data availability.

The next-generation-sequencing data obtained in this study are available under ArrayExpress accession numbers E-MTAB-7700 and E-MTAB-5122.
