# Supplementary figures and images for: Regulation of Antimycin Biosynthesis Is Controlled by the ClpXP Protease
Source: mSphere. 2020 Apr 8;5(2):e00144-20. doi: 10.1128/mSphere.00144-20 (PMC7142297; doi:10.1128/mSphere.00144-20)

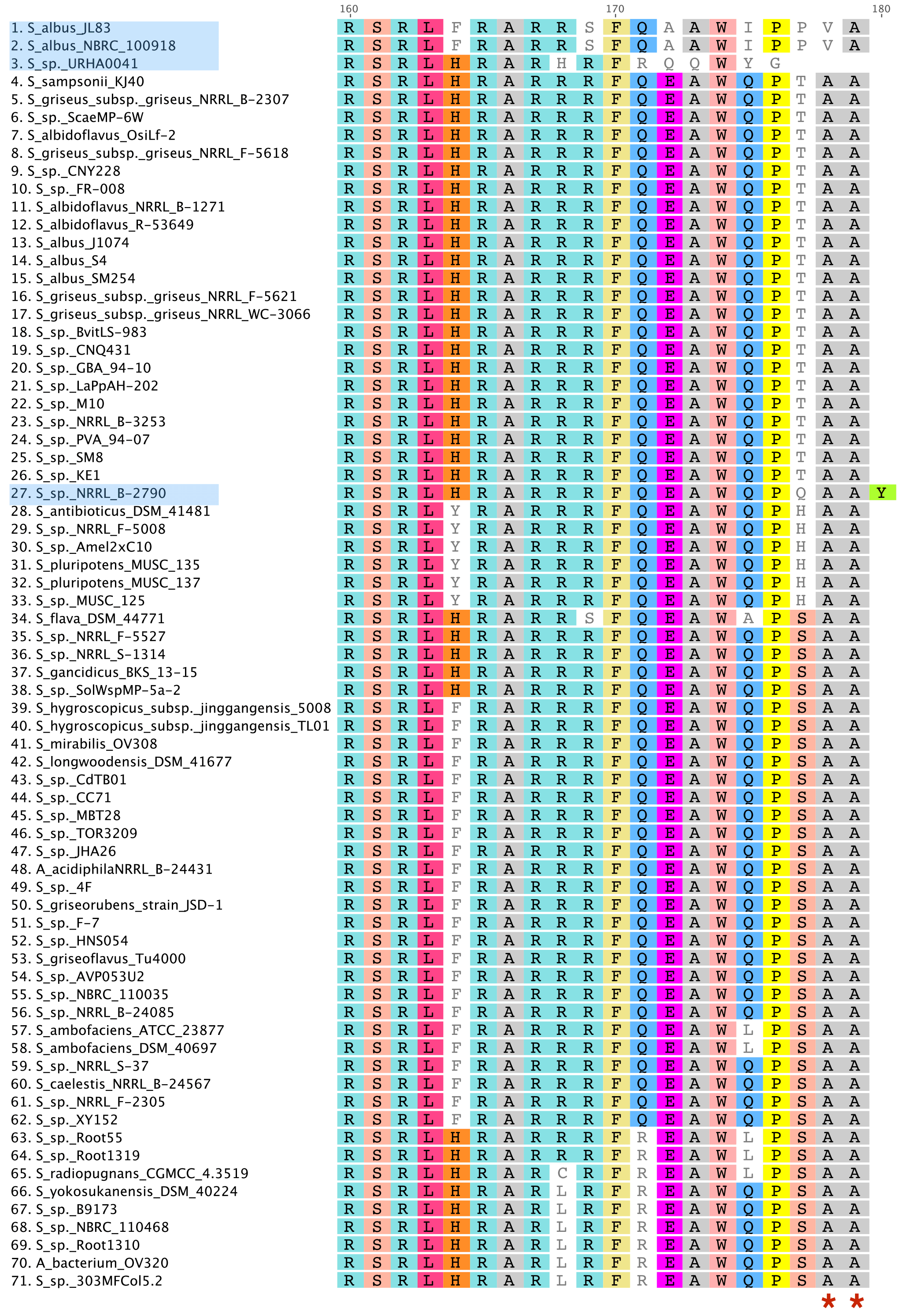

Supplement: FIG S1 [file mSphere.00144-20-sf001.gif]

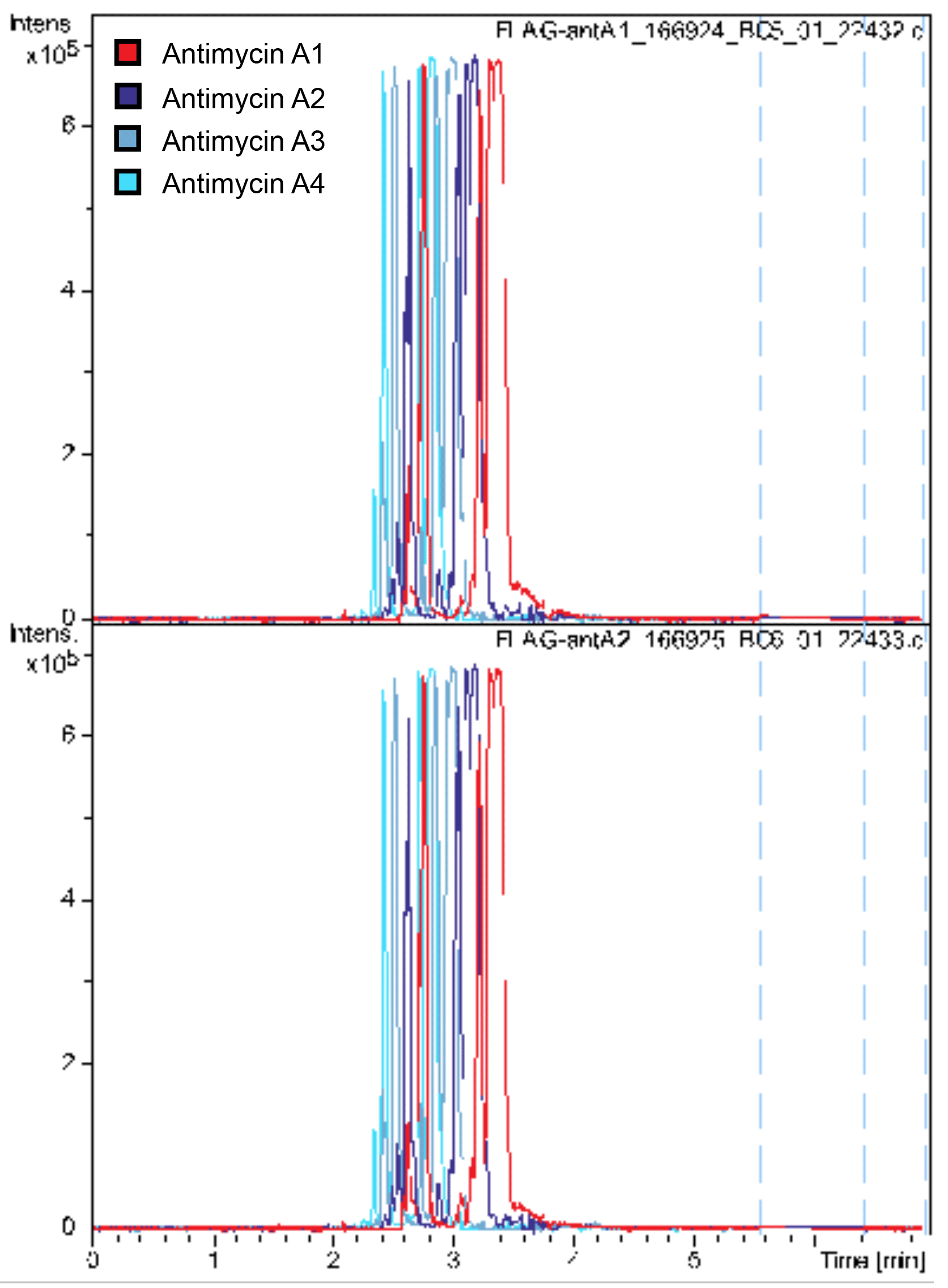

Supplement: FIG S2 [file mSphere.00144-20-sf002.gif]

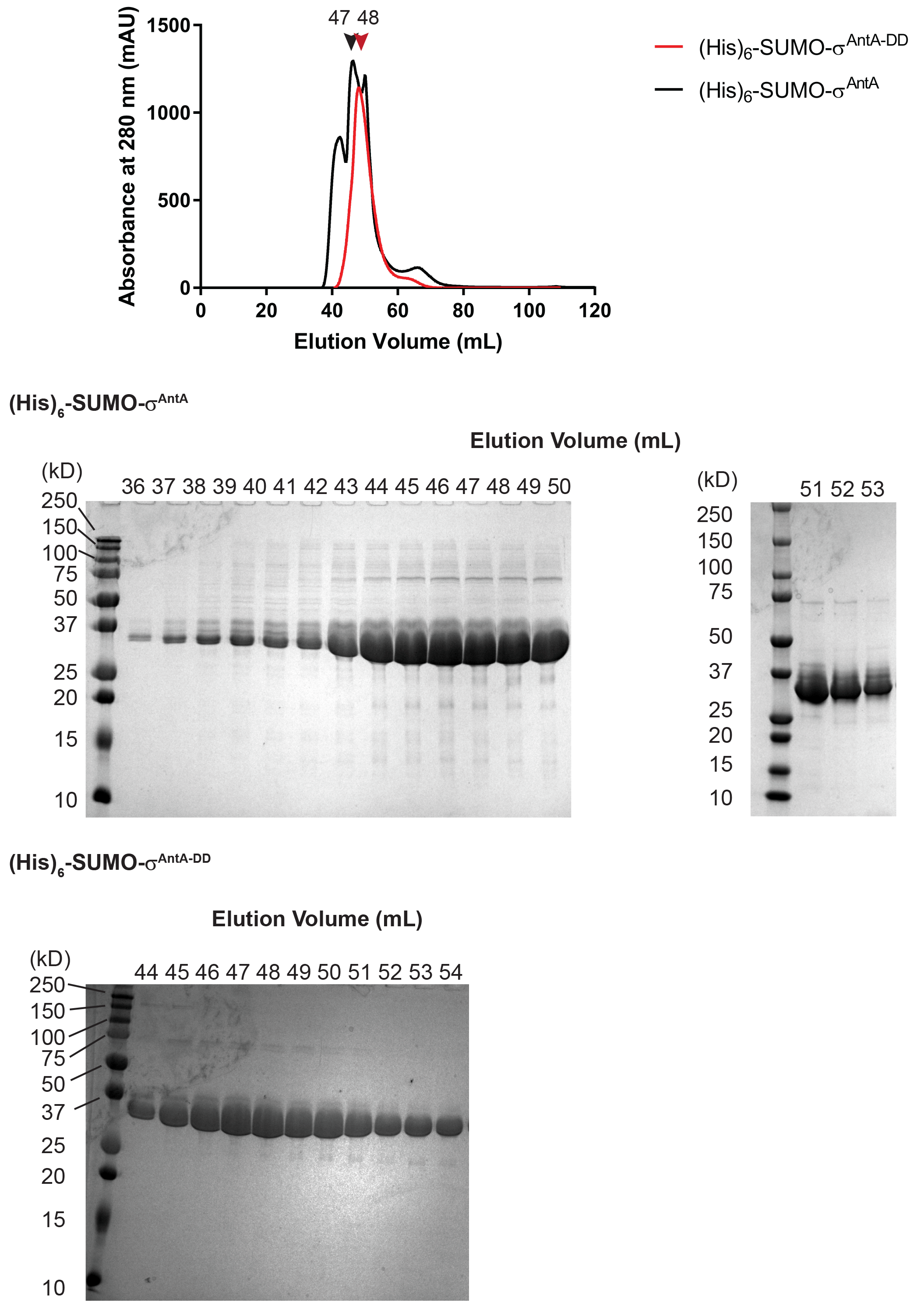

Supplement: FIG S3 [file mSphere.00144-20-sf003.gif]

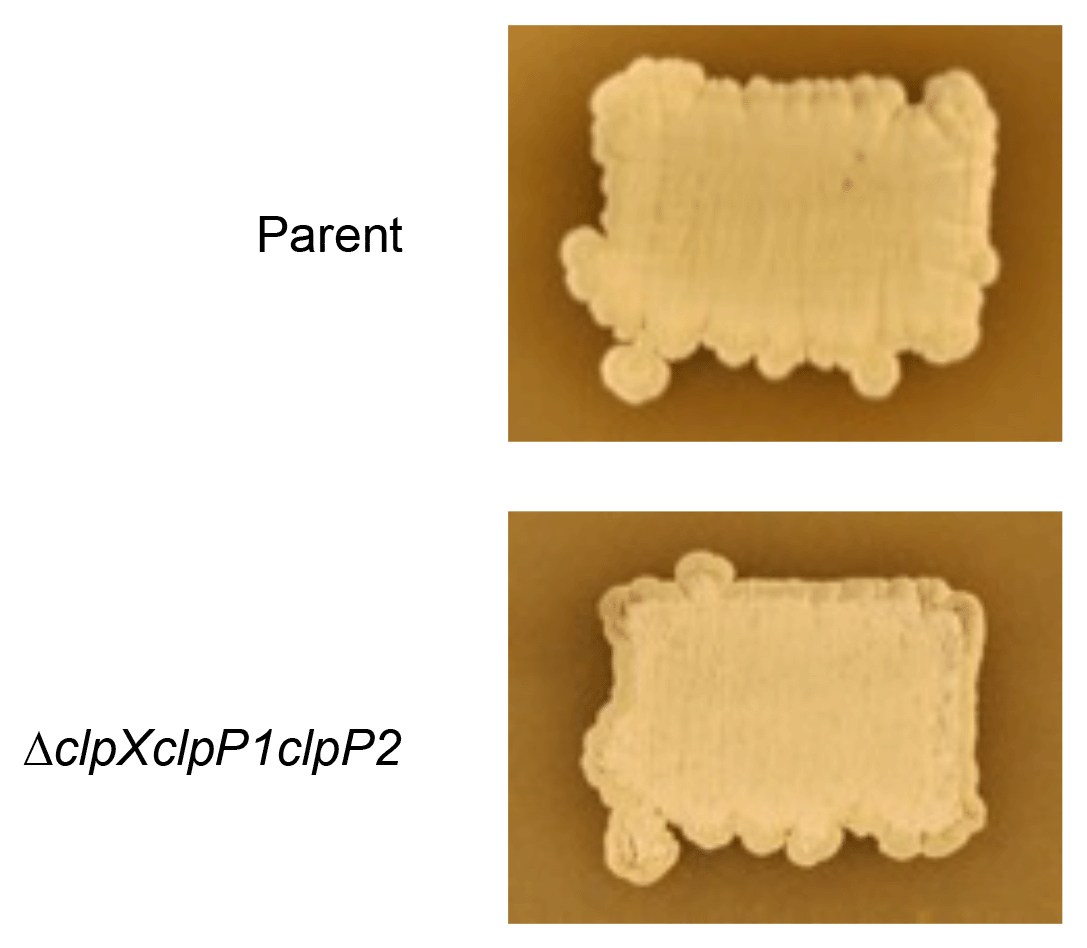

Supplement: FIG S4 [file mSphere.00144-20-sf004.gif]

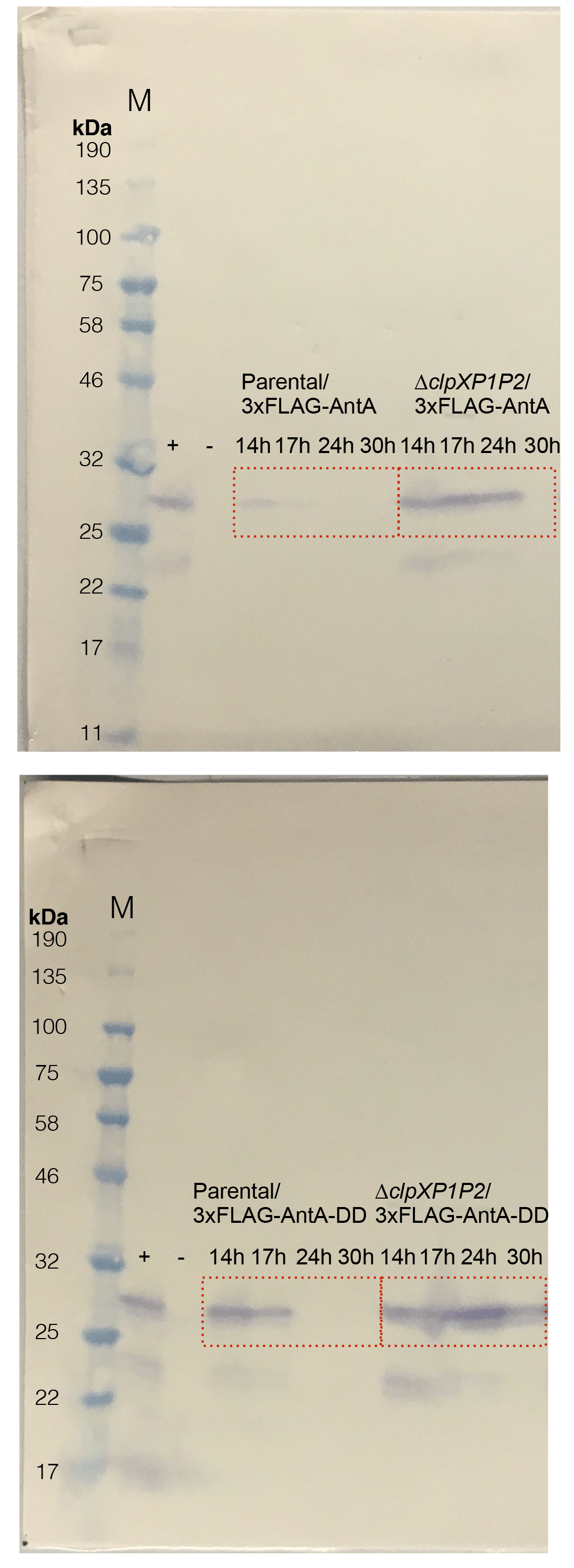

Supplement: FIG S5 [file mSphere.00144-20-sf005.gif]

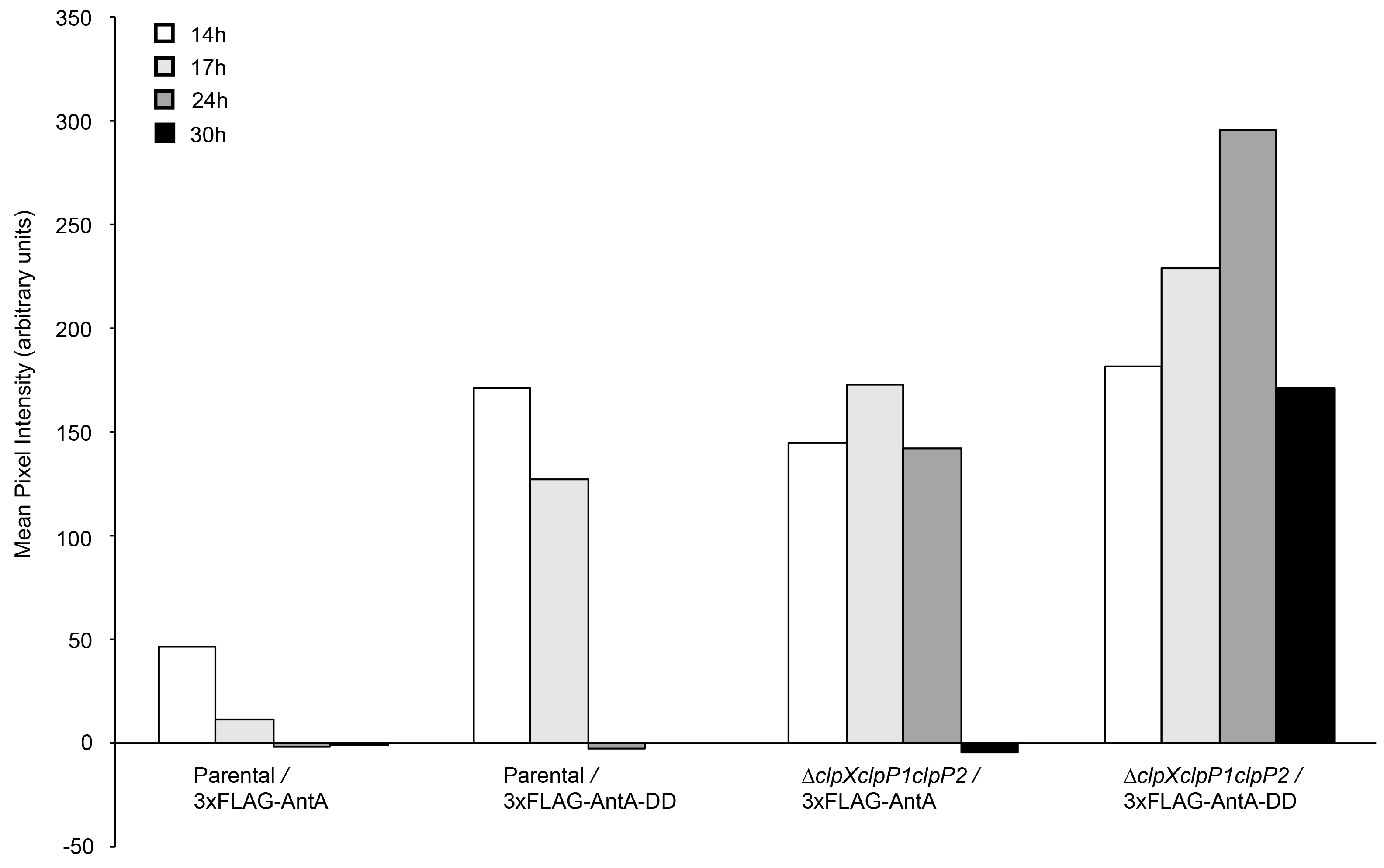

Supplement: FIG S6 [file mSphere.00144-20-sf006.gif]
