# Supplementary material for: Regulation of Antimycin Biosynthesis Is Controlled by the ClpXP Protease
Source: mSphere. 2020 Apr 8;5(2):e00144-20. doi: 10.1128/mSphere.00144-20 (PMC7142297; doi:10.1128/mSphere.00144-20)
Supplement: TABLE S1 [file mSphere.00144-20-st001.docx]

**Table S1. LCMS quantification of antimycin production**

|  | **Total antimycin peak area (arbitrary units)** | | |  |  |
| --- | --- | --- | --- | --- | --- |
| ***S. albus* S4 strain** | **Replicate 1** | **Replicate 2** | **Replicate 3** | **Average** | **SD*** |
| Parental (∆antall) *attB* ΦC31 cos213 | 17.24 | 15.3 | 17.24 | 16.59 | 1.12 |
| ∆antall∆*clpXclpP1clpP2 attB* ΦC31 cos213 | 18.71 | 13.11 | 14.88 | 15.57 | 2.86 |

* SD = Standard Deviation
