# Supplementary material for: Regulation of Antimycin Biosynthesis Is Controlled by the ClpXP Protease
Source: mSphere. 2020 Apr 8;5(2):e00144-20. doi: 10.1128/mSphere.00144-20 (PMC7142297; doi:10.1128/mSphere.00144-20)
Supplement: TABLE S2 [file mSphere.00144-20-st002.docx]

| **Table S2. Bacterial strains, cosmids and plasmids used in this study** | | |
| --- | --- | --- |
| **Name** | **Description** | **Reference or source** |
| ***Streptomyces* strains** | | |
| *S. albus* S4 | Wild type *Streptomyces albus* S4 strain | (1) |
| *S. ambofaciens* ATCC 23877 | *Streptomyces ambofaciens* wild-type strain | ATCC |
| ∆*antA* | *S. albus* S4 harbouring an *antA* deletion; Apr^R^ | (2) |
| ∆*antA*/pAU3-45-3xFLAG-*antA* | *∆antA* strain harbouring pAU3-45-3xFLAG-*antA* at the ΦC31 *attB* site; Apr^R^, Tsp^R^ | This study |
| Δ*antall* | *S. albus* S4 harbouring an unmarked deletion removing the entire antimycin BGC | (3) |
| ∆*clpXclpP1clpP2* | Δ*antall* strain with an unmarked mutation deletion removing the *clpX, clpP1, clpP2* operon | This study |
| ***Escherichia coli*** | | |
| XL10-Gold | General cloning host | Stratagene |
| ET12567 | Non-methylating host for transfer of DNA into *Streptomyces* spp. (*dam, dcm, hsdM*); Cam^R^ | (4) |
| GB05-red | Host for RecET recombination | (5) |
| Rosetta(DE3) | Host for heterologous protein production | Novagene |
| **Cosmids** | | |
| cos117 | Supercos1 derivative containing the *clpP1P2* and *clpX* genes; Carb^R^, Kan^R^ | This study |
| c117Δ*clpXP*::*aac(3)IV* | The c117-derivative with the *clpP1P2* and *clpX* replaced by the disruption cassette from paac+oriT; Carb^R^, Kan^R^, Apr^R^ | This study |
| **Plasmids** | | |
| patt-saac-oriT | PCR template for *aac3(IV)* oriT cassette used in REDIRECT PCR targeting system; Apr^R^, Amp^R^ | (6) |
| pAU3-45 | pSET152 derivative, integrates into *φ*C31 attachment site; Apr^R^ Tsp^R^ | (7) |
| pAU3-45-3xFLAG-*antA* | pAU3-45 derivative containing the *ermE**p-3xFLAG-*antA* cloned into the NotI and EcoRI sites; Apr^R^, Tsp^R^ | This study |
| pET23b-His-SUMO | pET23b with a SUMO tag cloned into the NheI-AgeI sites; Carb^R^ | (8) |
| pET23b-His-SUMO-*antA* | pET23b-His-SUMO derivative with the wild-type *antA* gene from *Streptomyces ambofaciens* ATCC 23877 cloned into the AgeI-HindIII sites; Carb^R^ | This study |
| pET23b-His-SUMO-*antA-DD* | pET23b-His-SUMO-*antA* derivative harbouring with point mutations changing the AntA C-terminal AlaAla to AspAsp; Carb^R^ | This study |
| pPDA | pSETNFLAG derivative harboring *antA* cloned into KpnI-EcoRI sites; Apr^R^ | This study |
| pPDD | pSETNFLAG derivative harbouring *antA* encoding A172D and A173D mutations cloned into KpnI-EcoRI sites; Apr^R^ | This study |
| pSET152 | *E. coli* – *Streptomyces* integrative shuttle vector, integrates into the ΦC31 attachment site; Apr^R^ | (9) |
| pSETNFLAG | pSET152 derivative with an *ermE**p cloned into the EcoRV-EcoRI sites; and an N-terminal 3xFLAG tag and multi-cloning site cloned into the NdeI-KpnI sites; Apr^R^ | (10) |
| pUWLint31 | *Streptomyces* vector for the expression of the ΦC31 integrase; pSG5 temperature sensitive *ori*; Tsp^R^ | (6) |
| pUZ8002 | Encodes conjugation machinery for mobilization of plasmids from *E. coli* to *Streptomyces*; Kan^R^ | (4) |
| Cam – chloramphenicol; Carb – carbenicillin; Kan – kanamycin; Apr – apramycin; Spr – spectinomycin; Hyg – hygromycin, Tsp - thiostrepton | | |

**References**

1. **Barke J, Seipke RF, Grüschow S, Heavens D, Drou N, Bibb MJ, Goss RJM, Yu DW, Hutchings MI**. 2010. A mixed community of actinomycetes produce multiple antibiotics for the fungus farming ant Acromyrmex octospinosus. BMC Biol **8:**109.

2. **Seipke RF, Patrick E, Hutchings MI.** 2014. Regulation of antimycin biosynthesis by the orphan ECF RNA polymerase sigma factor σ (AntA.). **PeerJ 2:**e253

3. **Fazal A, Thankachan D, Harris E, Seipke RF**. 2019. A chromatogram-simplified *Streptomyces albus* host for heterologous production of natural products. **Antonie Van Leeuwenhoek 8:**1–10.

4. **MacNeil, D.J., Gewain, K.M., Ruby, C.L., Dezeny, G., Gibbons, P.H., and MacNeil, T**. 1992. Analysis of *Streptomyces avermitilis* genes required for avermectin biosynthesis utilizing a novel integration vector. Gene **111:**61–68.

5. **Fu J, Bian X, Hu S, Wang H, Huang F, Seibert PM, Plaza A, Xia L, Müller R, Stewart AF, Zhang Y.** 2012. Full-length RecE enhances linear-linear homologous recombination and facilitates direct cloning for bioprospecting. Nat Biotechnol **30:**440–446.

6. **Myronovskyi M, Rosenkränzer B, Luzhetskyy A**. 2014. Iterative marker excision system. Appl Microbiol Biotechnol **98:**4557–4570.

7. **Bignell DRD, Tahlan K, Colvin KR, Jensen SE, Leskiw BK.** 2005. Expression of *ccaR,* encoding the positive activator of cephamycin C and clavulanic acid production in *Streptomyces clavuligerus,* is dependent on *bldG.* Antimicrob Agents and Chemother **49:**1529–1541.

8. **Wang KH Sauer RT, Baker TA**. 2007. ClpS modulates but is not essential for bacterial N-end rule degradation. Genes Dev **21:**403–408.

9. **Kieser T, Bibb MJ, Buttner MJ, Chater KF, Hopwood DA**. 2000. Practical *Streptomyces* Genetics. John Innes Foundation, Norwich, United Kingdom

10. **McLean TC, Hoskisson PA, Seipke RF**. 2016. Coordinate regulation of antimycin and candicidin biosynthesis. mSphere **1:**e00305–16.
