# Supplementary material for: Regulation of Antimycin Biosynthesis Is Controlled by the ClpXP Protease
Source: mSphere. 2020 Apr 8;5(2):e00144-20. doi: 10.1128/mSphere.00144-20 (PMC7142297; doi:10.1128/mSphere.00144-20)
Supplement: TABLE S3 [file mSphere.00144-20-st003.docx]

| **Table S3. Oligonucleotides used in this study** | | |  |
| --- | --- | --- | --- |
| **Name** | **Sequence (5’-3’)*** | **Description** |  |
| PBB001 | gtggcacgcatcggtgacggc | PCR: to identify the *clpX*-containing cosmid |  |
| PBB002 | tcaggccgacttctgctcgcc |  |  |
| PBB003 | gtgacgaatctgatgccctcc | PCR: to confirm presence of the *clpP1P2* operon on the cosmid |  |
| PBB004 | tcagactccggcgttgagctt |  |  |
| PBB034 | agacggcccggcgccgtcgtaagacgagcaggtggatacttccggggatccgtcgaccc | PCR: *clpXP*/*aac3(IV)*+*oriT* recombineering cassette |  |
| PBB035 | ggtggggcccttccgcgtgcgtctgccgggtgccggccctgtaggctggagctgcttcg |  |  |
| PBB015 | ccaaacggcgggcgcggaccc | PCR: to confirm the *clpXP* deletion |  |
| PBB018 | gacggaagggccccaccgcgc |  |  |
| RFS629 | tatata*ggtacc*aacaccgcgcacgaactgccc | PCR: to amplify σ^AntA^. Contains a KpnI site |  |
| RFS630 | tatata*gaattc*tcaggcggcggtgggctgcc | PCR: to amplify σ^AntA^. Contains an EcoRI site |  |
| RFS663 | tatata*gaattc*tcagtcgtcggtgggctgc | PCR: to amplify σ^AntA^, encodes A172D and A173D mutations. Contains an EcoRI site |  |
| SK221 | cgc*aagctt*tcacgccgcc | PCR: to amplify σ^AntA^. Contains a HindIII site |  |
| SK222 | tat*accggt*ggttccaccgtcagcgaactcc | PCR: to amplify σ^AntA^. Contains an AgeI site |  |
| SK232 | ggcatggctgccctcggatgattga*aagcttgcggccgc* | PCR: to mutagenize σ^AntA^. Contains HindIII and NotI sites |  |
| SK233 | *gcggccgcaagctt*tcaatcatccgagggcagccatgcc | PCR: to mutagenize σ^AntA^. Contains HindIII and NotI sites |  |

*Restriction sites are indicated by italics and non-homologous sequences are underlined
